# The Impact of Periodontal Inflammation on the Severity of Coronary Atherosclerosis

**DOI:** 10.7759/cureus.57653

**Published:** 2024-04-05

**Authors:** Esraa S Swilem, Nasima M Elkenany, Alaa S Algazzar, Nesma Youssef, Sara S Swilem, Eslam A Gendia, Ahmad S Swillem, Abeer A Elmalah, Zia Sabah, Tariq Rasool

**Affiliations:** 1 Faculty of Dental Medicine, The British University in Egypt, Cairo, EGY; 2 Department of Cardiology, Faculty of Medicine for Girls, Al-Azhar University, Cairo, EGY; 3 Department of Cardiology, Ahmed Maher Teaching Hospital, Cairo, EGY; 4 Faculty of Dental Medicine, Al-Azhar University, Cairo, EGY; 5 Faculty of Medicine for Girls, Al-Azhar University, Cairo, EGY; 6 Department of Medicine, King Khalid University, Abha, SAU; 7 Department of Medical Education and Simulation, University Institute of Computing, Chandigarh University, Chandigarh, IND

**Keywords:** periodontitis, cad, chd, pisa, coronary atherosclerosis

## Abstract

Introduction

Through plausible biological mechanisms, periodontitis causes systemic inflammatory burden and response, thus resulting in damage far beyond the oral cavity. Studies have demonstrated periodontitis to be a significant risk factor for coronary heart disease (CHD) and stroke. The larger the quantum of periodontal inflamed tissue, the greater the chances of periodontitis eliciting bacteremia and systemic inflammatory responses. Studies have reported that periodontitis and other common oral infections play an important role in the development of atherosclerosis. Therefore, the quantity of inflamed periodontal tissue assumes significance in determining the severity of atherosclerosis. Hence, this study investigates the impact of periodontal inflamed surface area (PISA) on the severity of coronary atherosclerosis.

Materials and methods

In this cross-sectional study, a total of 160 patients who presented at the department of periodontics of The British University in Egypt (BUE) from 1 January 2023 to 30 September 2023 were enrolled. Patients were only enrolled if they had undergone coronary angiography within the last six months, were less than 60 years of age, shared their previous medical history and coronary angiographic report, and gave informed written consent. Data on classic coronary risk factors and periodontal inflammatory status and angiographic findings were recorded and subjected to appropriate statistical analysis.

Results

The results revealed that the periodontal inflamed surface area (p = 0.002) apart from age (p < 0.047) and low-density lipoprotein cholesterol (LDL-C) (p < 0.001) is a significant independent predictor of the severity of coronary atherosclerosis.

Conclusions

The periodontal inflamed surface area is an independent predictor of the severity of coronary atherosclerosis.

## Introduction

Periodontitis, a chronic inflammatory disease primarily affecting the supporting tissues of the teeth, has been demonstrated to be caused by pathogenic periodontal bacteria [1,2]. In the majority of periodontitis cases, the bacterium *Porphyromonas gingivalis* is the causative agent [3,4], and in chronic cases, it leads to periodontal tissue wasting [5,6] due to stimulated inflammatory response and plaque formation [5]. However, periodontitis has also been reported to be a significant risk factor for a wide spectrum of diseases far beyond the oral cavity, such as cardiovascular diseases (CVD) and stroke [7-10]. Investigations studying the biological mechanisms by which periodontitis poses a risk for other systemic diseases hold that periodontitis induces an inflammatory burden by evoking bacteremia [11,12], systemic inflammatory responses [13,14], and cross-reaction, which results in autoimmune responses [15]. Traditionally, risk factors such as elevated serum cholesterol, decreased high-density lipoprotein (HDL), increased low-density lipoprotein (LDL), hypertension, diabetes, and smoking have been reported as the main precursors of coronary heart disease (CHD). However, these risk factors only account for 50%-75% of CHD burden [16,17].

There is concrete evidence that atherosclerosis is the main underlying cause of cardiovascular and cerebrovascular morbidity and mortality [18]. Earlier studies have shown that some common oral infections play a significant role in the development of atherosclerosis [19] and can lead to ischemic lesions in the heart, resulting in thromboembolic events and an infarction of the affected region [20]. There is primary evidence that infection by periodontal bacteria is significantly associated with heart diseases [21,22], and such infections play a direct role in the pathogenesis of atherosclerosis, which leads to thromboembolic events via sustained systemic challenges by inflammatory cytokines and liposaccharides [23]. Periopathogens *P. gingivalis* and *Streptococcus sanguinis* have been reported to induce platelet aggregation and activation, by expressing collagen-like platelet aggregation-associated proteins, which significantly contribute to the formation of atheroma and thromboembolic events [24]. In a study, periodontal pathogens *P. gingivalis*, *Prevotella intermedia*, *Bacteroides forsythus*, and *Aggregatibacter actinomycetemcomitans* were isolated from human carotid atheromas with 26%, 14%, 30%, and 18% prevalences, respectively [25]. Additionally, animal studies have reported the calcification of aortic atherosclerotic plaque in mice when exposed to *P. gingivalis*, and the length of exposure to these periopathogens is directly proportional to the amount of calcification [26].

Conventionally, the inflammatory status of periodontium is classified as per the clinical attachment level (CAL) index [27]. The CAL aggregates the probing pocket depth (PPD) and gingival recession. While gingival recession indicates past inflammation, the ongoing inflammation is reflected by elevated PPD. Thus, CAL indicates both the past and ongoing inflammation [28]. However, recently, a novel periodontal index, the periodontal inflamed surface area (PISA) index, which combines periodontal indices PPD, bleeding on probing (BOP), and plaque index (PI) in a single numeric variable, has been demonstrated to be capable of quantifying the periodontal inflammation and plaque accumulation status more efficiently compared to other periodontal indices [29]. However, despite our best efforts on the literature survey, we could not trace any investigation studying the association between periodontal inflammatory burden and the severity of coronary atherosclerosis.

Therefore, this study was undertaken to investigate the association between periodontal inflammatory burden and the severity of coronary atherosclerosis, with PISA as an indicator of the periodontal inflammatory status.

## Materials and methods

Study design and population

In this cross-sectional study, the subjects were recruited among the patients who attended the department of periodontics of The British University in Egypt (BUE) from 1 January 2023 to 30 September 2023. A total of 160 subjects conforming to the set inclusion criteria were included in the study.

Inclusion criteria

Patients were only enrolled if they were <60 years old, had undergone coronary angiography within the past six months, provided written informed consent, and shared their coronary angiographic report.

Exclusion criteria

Patients were excluded from the study if they had any orthodontic or prosthodontic devices and parafunctional habits, capable of aggravating or predisposing for periodontal diseases. Patients on periodontal treatments during the past year were also excluded from the study. The study was approved by the Ethical Clearance Committee of The British University in Egypt (approval number: 23-011) and was carried in line with the 2013 revision of the principles of the Declaration of Helsinki [30].

Assessment of periodontal inflammatory status

The assessment and quantification of inflamed periodontal tissue were done using the periodontal index: PISA [29]. Based on the clinical measurements of clinical attachment loss (CAL), recession, and bleeding on probing (BOP), PISA was calculated using the equations described by Hujoel et al. [31], as per the procedure described in another study [29].

Atheromatosis score calculation

The coronary atheromatosis score was calculated using the Gensini scoring system [32] as a score of 1 for ≤25% stenosis, 2 for 26%-50% stenosis, 4 for 51%-75% stenosis, 8 for 76%-90% stenosis, 16 for 91%-99%, and 32 for total occlusion. Additionally, based on the lesion’s position in the coronary circulation, the score of each lesion is multiplied by a factor of 5 if the lesion is in the left main coronary artery; 2.5 if in the proximal segment of the left anterior descending coronary artery; 2.5 if in the proximal segment of the circumflex artery; 1.5 if in the mid-segment of the left anterior descending coronary artery; 1.0 if in the right coronary artery, the distal segment of the left anterior descending coronary artery, the posterolateral artery, and the obtuse marginal artery; and 0.5 if in other segments. The individual coronary segment scores are summed to get the total Gensini score. The subjects were bucketed into three groups with regard to the tertile of the Gensini score: tertile I (Gensini score of <11 points), tertile II (Gensini score of 11-38 points), and tertile III (Gensini score of >38 points).

Conventional coronary risk factors

Data on age, gender, weight, height, smoking patterns, hypertension, and diabetes were obtained either from medical records or by an interview. Peripheral blood samples were also taken after an overnight fast for lipid profiling. Body mass index (BMI) was calculated using the adult BMI calculator by the Centers for Disease Control and Prevention (CDC), Department of Health and Human Services, United States of America [33].

Statistical analysis

All statistical analysis was performed using R [34] scripting language (R Foundation for Statistical Computing, Vienna, Austria) and the accompanying RStudio [35] (version 1.2.5033, Orange Blossom, Posit, Boston, MA). Sample size calculation was done based on the finite population correction for proportions version of Cochran’s equation [36]. Continuous variables were reported either as mean ± standard deviation (SD) or median (range), whereas categorical data were reported in numbers and percentages: n (%). Continuous variables following Gaussian distribution were compared using Student’s t-test, whereas continuous variables with non-Gaussian distribution were compared using the Mann-Whitney U-test. The chi-square test or Fisher’s exact test was used to compare sample proportions. The interrelations of PISA and conventional risk factors to coronary atheromatosis were assessed with stepwise logistic regression analysis. Coronary atheromatosis score (Gensini) was treated as a dichotomous variable with 35 as the cutoff point.

## Results

Table 1 presents the classical coronary risk factors and PISA score stratified by coronary atheromatosis score (Gensini) below and above the cutoff point of 35. Age, low-density lipoprotein cholesterol (LDL-C), and the PISA score were significantly high in patients having the highest coronary atheromatosis score. To assess whether the association between periodontal disease burden and coronary heart disease was independent of the other coronary risk factors, stepwise logistic regression analysis was done (Table 2). PISA (p = 0.002), age (p = 0.047), and LDL-C (p < 0.001) were independently associated with severe coronary atherosclerosis. In comparison, hypertension, BMI, total cholesterol, triglycerides, and HDL were not independent predictors of the severity of coronary atherosclerosis. Furthermore, excluding diabetics and patients on statins from the analysis did not alter the results. Additionally, no association was found between PISA scores and lipid, hypertension, or BMI status.

**Table 1:** Conventional coronary risk factors and PISA in the study participants stratified by coronary atheromatosis score (Gensini score) *Significant (p < 0.05) **Highly significant (p < 0.001) BMI, body mass index; HDL-C, high-density lipoprotein cholesterol; LDL-C, low-density lipoprotein cholesterol; PISA, periodontal inflamed surface area; SD, standard deviation

Risk Factor	Gensini Score Tertiles I + II (n = 93)	Gensini Score Tertile III (n = 67)	p
Age, mean ± SD	52 ± 5	54 ± 7	0.036*
Gender, mean (%)			
Male	84 (90%)	59 (88%)	0.689
Female	9 (10%)	8 (12%)	
BMI, mean ± SD	25.8 ± 3.1	26.6 ± 2.7	0.091
Hypertension, n (%)	52 (56%)	39 (56%)	0.801
Diabetes, n (%)	14 (15%)	12 (18%)	0.613
Total cholesterol, mg/dL (mean ± SD)	239.8 ± 46.4	243.6 ± 50.3	0.622
HDL-C, mg/dL (mean ± SD)	41.68 ± 8.3	39.15 ± 7.6	0.050
LDL-C, mg/dL (mean ± SD)	155.6 ± 24.5	164.2 ± 19.2	0.017*
Triglycerides, mg/dL (mean ± SD)	212.6 ± 23.6	219.3 ± 18.2	0.053
PISA, cm^2 ^(mean ± SD)	33.6 ± 4.2	36.2 ± 3.8	0.0001**

**Table 2:** Stepwise logistic regression (forward stepping) with PISA and other coronary risk factors as independent predictors and coronary atherosclerosis score (Gensini score) as the dependent variable *Significant (p < 0.05) **Highly significant (p < 0.001) PISA, periodontal inflamed surface area; HDL-C, high-density lipoprotein cholesterol; LDL-C, low-density lipoprotein cholesterol; NS, not significant

Independent Predictor	Regression Coefficient	Standard Error	p
PISA, cm^2^	0.41	0.13	0.002*
Age, years	0.14	0.07	0.047*
LDL-C, mg/dL	0.83	0.10	<0.001**
Total cholesterol, mg/dL	-	-	NS
HDL-C, mg/dL	-	-	NS
Triglycerides, mg/dL	-	-	NS

## Discussion

This study demonstrated a statistically significant association between the periodontal disease severity of coronary atherosclerosis. Here, the association between periodontal disease status as indicated by the PISA score, and coronary atheromatosis was of comparable magnitude as shown by the classical coronary risk factors; however, the association between serum triglycerides and coronary atheromatosis was smaller than expected.

The causality between periodontal infections and coronary atherosclerosis cannot be overlooked. An increasing number of investigations have linked periodontal disease to cardiovascular disease. DeStefano et al. [37] examined the association between periodontal disease and cardiovascular disease in the National Health and Nutrition Examination Survey (NHANES) I, wherein they followed 9760 study subjects for 14 years. They demonstrated that periodontal disease was strongly and independently associated with an increased risk of CHD. Interestingly, they demonstrated that males with periodontitis younger than 50 years of age had a 72% increased risk of CHD. Another study also showed that persons with radiographic evidence of periodontitis had a 0.5-2.8 times increased risk of developing CHD or suffering from a vascular event [38]. Arbes et al. [39] studied the link between periodontal disease and CHD in NHANES III and found an odds ratio (OR) of 3.8 (95% CI: 1.5-9.7) for having a heart attack with an increased severity of periodontal disease compared to no periodontal disease. In a meta-analysis of nine cohort studies, Janket et al. found that there was a 19% increased risk of cardiovascular and cerebrovascular events in persons with periodontal disease [7]. Likewise, in an interventional study, Noack et al. [40] demonstrated that in patients infected with periodontal pathogens, the C-reactive protein (CRP) levels were highly elevated, and CRP was an independent risk factor for cardiovascular disease (CVD). Ebersole et al. [41] have reported that interventions targeting periodontal diseases such as scaling, root canal treatment, and flurbiprofen result in reduced CRP levels one year post therapy.

The results of the present study support the hypothesis that periodontal disease has a role in the pathogenesis of cardiovascular disease. The mechanism behind these results is not fully understood, but both clinical and experimental studies indicate bacterial infections in general, and periodontal infections in particular have definite effects on endothelial cells [42-44], blood coagulation and thrombocytes [45-47], lipid metabolism [48], and monocyte-macrophages [49-51]. There is compelling evidence that infections by *Chlamydia pneumoniae*, *Helicobacter pylori*, periodontal bacteria, and *Cytomegalovirus* are associated with cardiovascular disease [22,21].

Study limitations

The study was a single-center study, and patients were clinically suspected of CHD at presentation and therefore had been on diet modifications and hypolipidemic and anti-ischemic medications. Therefore, the lipid status may not necessarily reflect the historical lipid status of the study participants; hence, the role of lipids may be interpreted with reservations.

## Conclusions

Compared to other periodontal indices, PISA is advantageous in describing periodontal inflammation and hence periodontal disease status, and it also facilitates multivariate regression analysis. Periodontal disease status as reflected by the PISA score is an independent risk factor for the severity of coronary atheromatosis. However, this finding does not prove causality but does value addition to the increasing body of knowledge linking periodontal disease status to CHD.
